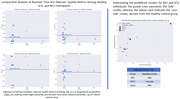# Conditionally Automated Vehicles and Cognitive Challenges: Assessing the Safety of Conditionally Automated Vehicles for Older Adults with Cognitive Challenges

**DOI:** 10.1002/alz.091988

**Published:** 2025-01-09

**Authors:** Gelareh Hajian, Bing Ye, Shabnam Haghzare, Jennifer L. Campos, Alex Mihailidis

**Affiliations:** ^1^ University of Toronto, Toronto, ON Canada; ^2^ KITE ‐ Toronto Rehailitation Institute, University Health Network, Toronto, ON Canada; ^3^ KITE ‐ Toronto Rehabilitation Institute, University Health Network, Toronto, ON Canada; ^4^ KITE Research Institute, Toronto Rehabilitation Institute ‐ University Health Network, Toronto, ON Canada

## Abstract

**Background:**

Driving cessation among people with cognitive impairments (e.g., Mild Cognitive Impairment; MCI) significantly impacts their independence and overall well‐being. Conditionally Automated Vehicles (CAVs) have emerged as a promising solution, potentially extending the safe driving years of at‐risk drivers. However, the safety of using CAVs for individuals with cognitive impairment remains underexplored. Individuals with Subjective Cognitive Decline (SCD; a potential early biomarker for later clinical decline), may also be at‐risk for difficulties managing complex behaviours such as driving. Therefore, this study aims to evaluate the driving performance of older adults with normal cognition, SCD, and MCI to assess their ability to safely transition between automated and manual driving modes using a simulated CAV.

**Method:**

Preliminary data from 17 cognitively healthy older adults, 2 with SCD, and 1 with MCI have been collected. Participants performed conditionally automated driving tasks using a high‐fidelity driving simulator under varied environmental conditions (daytime, nighttime), road geometries (straight, curved), with varying speed limits. A detailed analysis of driving safety, focusing on key metrics such as takeover reaction time and quality (lane centering, steering variation, and acceleration variability) was conducted. A performance baseline was established from the healthy group, segregating them into ‘safe’ and ‘less safe’ categories using a clustering method. The driving performance of participants with SCD and MCI was compared to the control group. By calculating Euclidean distances from cluster centroids we identified the most representative cluster for each individual.

**Result:**

The clustering technique successfully distinguished between ‘safe’ and ‘less safe’ drivers within the cognitively healthy group, outperforming traditional manual methods. This baseline then enabled a comparative assessment for participants with MCI and SCD, demonstrating the ability of our approach to assess whether individuals with SCD and MCI were able to safely perform takeover requests.

**Conclusion:**

This study provides valuable insights into the safety of individuals with cognitive challenges to use CAVs. Our ongoing research aims to expand the participant sample, further explore the relationship between cognitive abilities and driving performance in CAVs, and ultimately develop a methodology to assess and predict the safety of using CAVs for older adults with cognitive impairment.